# A co-produced service evaluation of ethnic minority community service user experiences of a specialist mental health service during the COVID-19 pandemic

**DOI:** 10.1186/s12913-023-10115-4

**Published:** 2023-10-17

**Authors:** Kiranpal Kaur, Daniel Mutanda, Palo Almond, Aparajita Pandey, Paris Young, Tony Levitan, Anna-Marie Bibby-Jones

**Affiliations:** Sussex Partnership NHS Foundation Trust, Worthing, UK

**Keywords:** Ethnic minority, Service evaluation, Mental health, COVID-19, Service improvement, Service user narrative, Online therapy, Access barriers, UK, Pandemic

## Abstract

**Background:**

For ethnic minority communities in the UK, the COVID-19 pandemic amplified existing health inequalities and created other consequential disadvantages like increased vulnerability to COVID-19, higher rates of hospital admissions, increased mortality and poorer mental health outcomes. While longer-term impacts of COVID-19 are considered, it is crucial for NHS mental health services to understand the specific barriers and needs of ethnic minority communities to provide consistent and equitable access to mental health services. These aspects were the focus of a service evaluation of a Sussex-wide mental health service conducted in co-production with experts-by-experience, public members, health professionals and researchers from ethnic minority communities.

**Methods:**

Co-designed creative workshops (n = 13) and semi-structured qualitative interviews (n = 13) were used to explore experiences of accessing specialist mental health services during the COVID-19 pandemic. Participants were: Sussex Partnership NHS Foundation Trust (SPFT) service users recruited between October 2021 and January 2022; aged 16+; from ethnic minority community backgrounds. Data was analysed using Thematic Analysis.

**Results:**

The analysis yielded five overarching themes contextualising service users’ experiences: (1) limited awareness of SPFT mental health services; (2) effects of COVID-19 in gaining access to SPFT; (3) SPFT reaching out to ethnic minorities; (4) being supported, 4a) hiding my mental health status from friends and families, 4b) lack of ethnic diversity in services, and 4c) better provision of information and support services, (5) relationship between childhood experiences and current mental health. These findings led to seven key recommendations for future service developments within SPFT.

**Conclusions:**

Although this evaluation was set in the context of COVID-19, findings have highlighted specific mental health service needs for ethnic minorities that are applicable beyond the confines of the pandemic. Many benefited from online sessions seen as more inclusive. Mental health advocates, outreach and joint working with communities could help further reduce stigmatising attitudes and improve engagement with mental health services. Improved service awareness of the impact of childhood or historical traumas experienced by ethnic minority communities on current mental health, the role of cultural awareness training and availability of culturally adapted therapies is also needed. Many service improvement recommendations provided could impact all service users.

**Supplementary Information:**

The online version contains supplementary material available at 10.1186/s12913-023-10115-4.

## Introduction

In March 2020, the COVID-19 pandemic was declared a worldwide emergency by the World Health Organisation and caused multiple unprecedented changes to society. The UK Prime Minister at the time, Boris Johnson, ordered a national lockdown instructing the public to “Stay at home, Protect the NHS and Save lives” [1, 2]. The public were not allowed to leave their homes unless it was thought essential [3]. Since then, the COVID-19 pandemic has caused multiple lockdowns, which have resulted in restricted delivery of UK healthcare services and made access more difficult [3, 4]. Many individuals, with and without pre-existing mental health conditions, experienced a decline in their mental health [5] leading to soaring demands for NHS support [6]. Poorer mental health outcomes were linked to COVID-19-related factors such as financial difficulties, infection and mortality rates, and home-schooling [7, 8].

Even prior to the COVID-19 pandemic, mental health services were already facing significant challenges. These included bed shortages, high service demands, frontline workers struggling with burnout, and staff shortages [9]. These difficulties were further compounded by inadequate resources and a growing population in need of mental health support. Globally, mental health services have been recognised as of poorer quality than physical health services with greater inequality for ethnic minority communities, asylum seekers, refugees, migrants and those who face racial discrimination [10, 11]. The arrival of COVID-19 exacerbated existing problems, leading to a more severe situation where service users may not have received the necessary support when needed most [12–15].

For many UK individuals from ethnic minority communities, lack of timely support has been their reality even prior to the pandemic [16]. The health inequalities experienced by ethnic minority communities are well documented and extend back decades [16, 17] Historically, the mental health burdens specific to minoritised individuals have been associated with socio-economic factors such as housing conditions [18]; stigma-related cultural influences [19–21]; long impacting trauma born from long term historical events such as genocide, slavery and forced migration [22, 23]; and, racism and discrimination [24]. Alongside this, many minoritised people do not access services due to internalised stigma about mental health services [25] and others are unaware of the help available [20]. Among those who do encounter mental health services, minoritised people experience poorer outcomes such as higher rates of detention, higher use of restrictive practices, poorer care, and reduced long-term recovery rates [21, 25, 26]. Meanwhile, it is evident that COVID-19 has had a disproportionately negative impact on the mental health of people from ethnic minority communities [15, 27–29].

Aside from the historical and structural burdens described above, some ethnic minority communities also have a higher risk of contracting COVID-19, higher rates of hospital admissions, a greater need for intensive care, and increased mortality [30–33]. Researchers [33] have highlighted 6 key pathways which have potentially led to the unequal impact of COVID-19 on ethnic minority communities through differentials in: virus exposure, vulnerability to infection, health consequences, social consequences, effect of pandemic control measures, and adverse consequences of control measures. These pathways have been corroborated by others [24, 33–35]. We now have an improved understanding of the anxiety and fear associated with COVID-19, and its impact on pre-existing mental health difficulties and access to services [24]. As we consider the longer-term impacts of COVID-19, NHS mental health services need to do more work to understand the specific needs of ethnic minority communities and provide consistent, timely and equitable access.

This paper describes the process and findings of a co-produced service evaluation to explore ethnic minority community service users’ experiences of accessing a large regional specialist mental health service during the COVID-19 pandemic. It also describes service users’ recommendations for future service development to support equity of access and mental health outcomes for ethnic minority communities. The co-production team was composed of experts-by-experience, public members, health professionals and researchers from different ethnic minority community backgrounds. The team followed guidance on the principles of co-production by the National Institute for Health and Care Research (NIHR) [36]. This included remunerating experts-by-experience and public members for their involvement, joint decision making and allowing for extra time. The team met monthly via Zoom from January 2021 to May 2022 to discuss study design and conduct. An evaluation of the experiences of ethnic minorities with a learning disability was also planned using proxy methods. However, parents and carers indicated that additional COVID-19 related stresses hindered them from participating and so this aspect was abandoned.

## Methods

### Design

The aim of this study was to explore ethnic minority community service users’ experiences of mental health services during the COVID-19 pandemic using co-production. A mix of creative workshops and semi-structured qualitative interviews were used for data collection; the frequency, duration, structure, and content of these sessions was decided by the co-production team. The interview guide for the qualitative interviews was informed by information collected through the creative workshops (delivered online, in three parts and using a focus-group style) exploring individual service user journeys. At the point of recruitment to the project, service users were given the option of attending the creative workshops, individual interviews, or both.

This service evaluation was registered with the Sussex Partnership NHS Foundation Trust (SPFT) Quality Improvement Team on 12/04/21 and approved by SPFT Information Governance on 22.07.21 (ref DPIA232SPFT).

### Participant recruitment

Participants were service users recruited from SPFT, a large specialist mental health and learning disabilities service. In 2021, national Census data indicated SPFT served 1.7 million residents in Sussex with 9% from ethnic minority community groups [37]. Recruitment was conducted between October 2021 and January 2022 with the following inclusion criteria: aged 16 or over; from a self-ascribed ethnic minority community background; and, had accessed SPFT services since March 2020. To maximise inclusivity, non-English speaking service users were encouraged to participate, and interpreters were available on request.

Recruitment materials (posters and leaflets) were designed in consultation with ethnic minority community people with mental health problems. These included illustrations of people from different ethnic backgrounds (see Supplementary Materials 1 for the poster; members of the co-production team used personal and community knowledge to identify the best places to display the posters). The materials were used to advertise the project through social media (Facebook and Twitter). SPFT lead mental health practitioners, who had established relationships with service users from ethnic minority communities, were assisted by the co-production team to identify eligible people on their caseload. Practitioners then contacted potential participants directly and shared the recruitment materials. A member of the co-production team also attended an ethnic minority community support group to advertise the study.

Volunteers were invited to an initial one-to-one telephone call with a member of the co-production team who discussed project details, confidentiality, anonymity and the right to withdraw from the project at any time without any effect on their care by SPFT. An opportunity was given to ask questions and then informed consent was gained and demographic information was collected (e.g. age, sex, employment status, service access, and length of time using the service). All service users and public members taking part in any aspect of the project were remunerated for their time. This was either by following the Trust’s Service User & Carer Payment Policy for involvement work guidance, or by receiving £15 for each creative workshop or qualitative interview attended.

### Data collection

The topic guide for the qualitative interviews (shown in Supplementary Materials 2) was informed by findings from the creative workshops. The creative workshops were collaboratively designed by the co-production team’s experts-by experience, the peer researcher (KK) and healthcare professionals. They were delivered by a healthcare professional from the co-production team and mental health peer facilitators recruited just for the workshop (i.e. they did not attend co-production meetings). During the creative workshops, participants were invited to illustrate their experiences of using SPFT services using different mediums e.g. art, poetry and/or writing. The facilitators helped participants start and develop their creative pieces during the sessions. Pieces were completed outside of the sessions. During the sessions, participants described how they felt about the process and what their creative pieces represented. For those consenting, creative pieces were collated and published in a book for the participants to own. Workshops took place in November and December 2021. Thirteen people took part in the workshops in groups of four/five with each delivered over three consecutive weeks.

Semi-structured one-to-one interviews, conducted by KK, were used for exploring the lived experiences of service users accessing SPFT mental health services during the COVID-19 pandemic. This method allows for new emerging themes to arise using open-ended questions [38].

### Data analysis

With informed consent, interviews were audio recorded, transcribed and then thematically analysed using Braun & Clarke’s methods [39] by KK. Identifying information such as names and locations were omitted and pseudonyms were used instead. Transcript coding was guided by set *cycles* as defined by Saldaña (2009) [40]. The first cycle in the coding process refers to the initial coding stage, which involves constant rereading in a descriptive manner where language is identified at a textual level. The second cycle involves analytically indexing codes which were appropriate to the study’s questions, aims, and objectives. Codes were then prioritised and synthesised which culminated in the generation of themes. Data saturation was achieved when no new themes emerged from the data. Themes were checked and confirmed by PA (a public member of the co-production team) and then discussed with the co-production group. These were later confirmed with participants at a celebration and dissemination event in May 2022. Their suggestions were incorporated into the final set of recommendations.

## Results

Thirteen people took part in the creative workshops and were: African (1), Bangladeshi (2), Black Caribbean (2), British Indian (3), Filipino (1), Mixed Heritage (1), Polish (1), White & Black African(1) or self-ascribed as another ethnicity (1). Thirteen service users (including six from the creative workshops) were recruited for the semi-structured interviews between January and February 2022. Interviewees’ ages ranged from 24 to 48 years and included 10 females and three males. Table 1 shows interviewees’ ethnic backgrounds and the pseudonyms used to humanise their lived experiences whilst protecting their identity. Twelve interviews were conducted via the online platform Zoom and one interview was conducted via telephone (all interviews were conducted by KK). Interviews lasted approximately 60 or 90 min if an interpreter was present.

**Table 1:** Service user pseudonyms and ethnic background

Pseudonym	Ethnic group (self-ascribed)
Eloise	Mixed Race, White and Black African
Zara	Arabic
Arti	Mixed Race, Indian and White
Gehna	Bangladeshi
Gulya	Middle Eastern
Anita	Black Caribbean
Zoran	Kurdish
Yuvraj	British Asian Indian
Anne	Black British
Faiza	Pakistani
Sekani	African
Nenita	Filipino
Lou	Other ethnicity

Thematic analysis of the fully transcribed 13 interviews found five overarching and recurring themes and subthemes which are displayed in Table 2.

**Table 2:** Overarching themes and subthemes found in the analysis

Themes	Subthemes
1. Limited awareness of SPFT mental health services	
2. Effects of COVID-19 on gaining access to SPFT	
3. SPFT reaching out to ethnic minorities	
4. Being supported	4a. Hiding my mental health status from friends and families
	4b. Lack of ethnic diversity in services
	4c. Better provision of information and support services
5. Relationship between childhood experiences and adult health	

### Themes

#### Limited awareness of SPFT mental health services

This theme captures service users’ experiences of accessing SPFT mental health services. Many service users expressed they were unaware of SPFT mental health services and therefore unable to seek help sooner in their mental health journey:

Zoran: It’s because they [service users] don’t know where to start, who to go… I wasn’t aware of SPFT, it was the [community group] that told me about SPFT services.

Zara: I only found out from my friend as she talks to a lot of people…she’s socially more like active so she knows more people she’s more educated.

Faiza: the educated people they know that sometimes they need help and they come, but the people like that who are not very educated, they could call housewives they don’t have awareness.

Three service users, from different ages and ethnic minority community groups talked about their experience attempting to access SPFT mental health services. They seemed to have little awareness of the services SPFT has to offer. Zoran and Zara both learnt about SPFT from an external source. Interestingly, Faiza reflects upon members of her community and the concept of the “simple housewife” and how people from her socio-economic group are less informed and hence lack awareness about the existence of these services. Faiza also described the “simple housewife” as a woman who is under the control of her husband.

Faiza: he [husband] knows he can’t stop me, ok because [names her job occupation] so he knows that she knows her rights, he can’t stop me, right, somewhere he has in mind because she is studying…she can do any action if I cross my boundaries right…, but for a lady their husband knows she knows nothing she can’t do anything, he won’t allow it, he might not allow.

According to Faiza, in her culture, women who are often controlled by their husbands are referred to as “simple housewives.“ Faiza shared how her education had helped her break free from such control. Faiza goes onto say that due to her training and qualifications, her husband cannot deny her medical care. She also highlighted that women who were considered “simple housewives” and lacked education were vulnerable to such control and may not have knowledge about mental health services.

#### Effects of COVID-19 on gaining access to SPFT

The pandemic affected service-user access to many mental health services around the UK. Following Government restrictions, many face-to-face appointments were halted. However along with many other health care organisations, SPFT moved to offering virtual access to therapy sessions. Anne, Lou and Nenita talked about the benefits of this decision and how it positively impacted their mental health outcomes.

Anne: we have had groups online and they are still continuing online, which is good.

Lou: one good thing came out of it [COVID-19], but it made things more accessible for people who can’t leave home because everybody couldn’t… I think was a learning point for mental health services to realise the impact that you know, social anxiety can have and yeah, to make things more accessible.

Nenita: with mental health it improved coz of online groups so those ones I mentioned with the [SPFT service] I accessed during COVID, it was so easy to do that and I think what they do is brilliant.

Online therapies and treatments were fairly limited prior to the COVID-19 pandemic and it took a pandemic to extend these services more widely. Whilst it is not known how clinical staff were trained or prepared to provide therapy online, it is clear some service users were grateful for this service. Online sessions were seen to benefit those who are unable to leave their homes or have work commitments for instance.

#### SPFT reaching out to ethnic minority communities effectively

Service users talked about ways in which SPFT could reach out more to ethnic minority communities to offer mental health support. A common theme throughout this evaluation was that ethnic minorities yearned to be understood. Service users talked about the historical trauma [22, 23] they have faced, which has left a mark on their outlook on life. For Anne, Yuvraj, and Zoran, historical trauma has become a part of their “identity”. Some felt strongly that consultations should go beyond “touching surface” [Zoran, Yuvraj, Gulya] and for cultural traumas to be acknowledged.

Yuvraj: my family is still suffering from the 1971 genocide of East Pakistan. That’s a fact. It’s a family trauma. It had a significant impact on us.

In communities where mental health remains a taboo subject, individuals in need of help may not come forward. Therefore, service users have recommended that SPFT go out to ethnic minority communities with the specific aim to cross cultural barriers. Yuvraj was encouraged to seek mental health support by an advocate who he mentions was “armed with the facts”. Even though from another ethnic minority community background, the advocate still understood Yuvraj’s condition and his family dynamics. These were potential factors which “a Westerner wouldn’t understand”. Yuvraj recommends more culturally competent mental health advocates to help break the barrier between service user and service provider.

Yuvraj: It was a mental health advocate who I felt knew something about my background, and where I had come from, and why I drank and used drugs the way I did and they were able to put me to the resources … he was armed with the facts, frothy emotional appeal from someone.

is not going to do it, you either know my background or you don’t…a Sussex Health promotion officer or an ideal one should establish liaisons with the mosques, the madrasas, the temples, the gurdwaras.

#### Being supported

This overarching theme of being supported was extensive and so was split into three subthemes: hiding my mental health status from friends and families; lack of diversity in services; and better provision of information and support services.

4a) Hiding my mental health status from friends and families.

Many reported feeling unable to disclose their mental health difficulties to their families or others in fear of the cultural stigma that is often attached.

Anne: They don’t … don’t know how ill I am because I try and hide that aspect.

Faiza: I don’t I I never have any session in presence of my husband no, no, I don’t want to tell him he know he knows that I go to psycho when when I was having time to talk he used to drop me there but he never knew that why, why because you know if he will come to know he’ll definitely want to tell his mum or sister at any point or at some point, at some point so I never disclose that why I’m going I keep it personal very personal.

The literature suggests that distancing oneself from others to avoid the stigma related to mental health is a common reaction among people with mental health diagnoses [41, 42]. Many service users in the project reported adopting this reaction to avoid feeling judged by their families, evade likely negative consequences, and maintain positive social identities. For example, Anita is described by her family as “the light and soul of the party”, however, she worried this perception could be tainted by unsolicited views of her mental health diagnosis. Faiza mentions she keeps the details of her therapy session private from her husband out of fear that he will share them with his mother, who in turn will spread the information to others. This illustrates the pressures some experience trying to live up to other people’s expectations possibly at the expense of their mental health.

4b) Lack of ethnic diversity in services.

Outside of SPFT, Yuvraj attends a recovery group, one which he defines as a “community.” His experiences shed insight into the benefits of peer-to-peer support as many service users did not have this at home. In addition, many implied that they wanted to be part of groups where they may feel more understood being from an ethnic minority community background. Anne, Anita, and Arti talked about being somewhat distanced from group peers due to the lack of cultural or ethnic diversity.

Anne: it’s kind of really been just me. You know, me as a black woman.

Anita: I guarantee I’ll probably be the only black person there. And then actually feel different again.

Arti: there was only like a few people of, who were non-Caucasian.

4c) Better provision of information and support services.

Service users who were receiving therapeutic support and/or were seen in Accident & Emergency were sometimes given SPFT information booklets/packs and then sent away. While some appreciated the content, many were overwhelmed by the information they were expected to absorb and make use of when they were also experiencing mental illness or a crisis.

Eloise: when we are in those moments, the last thing you can do is read it, read or understand anything you are reading, you have to read the same three words like over and over again, until it gets through to your mind.

Nenita: I would have preferred to have gone through that with someone rather than being given the task to do.

There is perhaps a need to improve SPFT practitioners’ awareness of when and how to appropriately provide resources to service users. Sharing a similar experience to Eloise, Nenita made the recommendation of being able to go through these materials with a practitioner. This would have implications for staff training on when and how to share information, especially in emotionally heightened situations such as during A&E attendance.

Service users who are unable to speak English often relied on interpreters to convey their message and translate information back to them. According to service users, accents, dialects, and cultural awareness are essential components which make up a suitable interpreter. For Gehna, having an interpreter to speak on her behalf is important otherwise she “does not get the service”. However, if a barrier to service access is the impact of accents and dialects, service users are further disadvantaged when accessing SPFT.

Zoran: even though we speak the same language, there are words we don’t understand each other.

Zara: there are always interpreters but they’re all different accents and I have problems understanding them and they have problems understanding me.

Gehna, Zoran and Zara are from different ethnic minority community backgrounds, however, they have emphasised that accents and dialects are often overlooked in terms of their significance when booking an interpreter. To put this into context, Arabic is spoken in over 22 countries which means there will be over 22 different accents and dialects [43].

#### Relationship between childhood experiences and current mental health

Whilst reflecting on their experiences during the pandemic, some service users disclosed trauma caused by being bullied during childhood and some described links to the present. Nenita expressed how words can “stick” to you and “can have a massive effect on an individual”. Similarly, Arti also reflected on her experiences of being bullied throughout her childhood describing children being racist and often calling her names at school. Lou talked about this as an issue currently affecting her children and described having to explain to them that the “joke” they experienced at school was racism.

Lou: they’re half Chinese and they were told that they brought the virus in… it was seen as a joke.

For Anne, her trauma of being physically and emotionally abused when she was younger was triggered by the Black Lives Matter protests which occurred during the early months of the pandemic.

Anne: the whole sort of BLM thing made me feel really anxious cause I’ve had so much abuse, not just [where I live now], but when I was younger, it kind of triggered me a lot.

It is well known that bullying can lead to mental health difficulties in victims [44]. What is less known is to what extent children are bullied and how this has affected their mental health in adulthood. Eloise therefore recommends the provision of a school-based mental health service creating “a safe place” to talk in an environment where they spend a lot of their childhood.

## Discussion

It is clear that SPFT service delivery was seriously affected by the COVID-19 restrictions. One service user recalled how it took two lockdowns to be seen by an SPFT mental health service. However, issues such as these were not unique to SPFT [45] and were driven by hospital staff absences mirrored nationally. In January 2022, 40% (28,000) of UK staff were reportedly off work due to COVID-19 [46, 47]. This is consistent with the SPFT Annual report (2021) [48] which highlighted more sicknesses and periods of self-isolation among staff during lockdown periods.

While many face-to-face therapy sessions were halted, SPFT quickly delivered therapy sessions through online platforms. Service users generally viewed the online therapy received as positive (other delivery modalities were not mentioned). This contradicts existing literature which regarded remotely delivered therapy as a negative experience [49, 50]. Nevertheless, other findings have shown that online interventions are effective and beneficial to mental health [51, 52]. Service users recommended this approach be used more frequently noting its inclusivity for those physically unable to travel or with anxiety disorders. For those with challenges juggling therapy with work commitments, an online solution is a helpful option which can potentially improve engagement [53, 54]. However, existing literature has shown that for some, home is not a “safe space” and therefore receiving therapy in their home is not feasible [55, 56], thus possibly leading to further disadvantage in an already vulnerable group. Offering a choice between remote and face-to-face therapy, as a blended approach, would be beneficial to those unable to attend in-person or unable to talk privately in their home [57].

Consistent with findings in the literature [58], socio-demographic factors such as education were found to impact service-user access to mental health services. Therefore, service users have recommended raising awareness among those from different socio-demographic groups (education, social class, and occupation).

Many service users reported facing stigma about their mental health difficulties and felt unable to disclose associated struggles to their families. This is consistent with existing literature which identified that many communities try to hide diagnoses [59, 60]. Furthermore, those who seek help may not be receiving social support from home which may lead to poorer mental health outcomes [61]. It is important for mental health services to better understand the existing barriers and social contexts within communities, to act upon this, and find ways to reach out to groups and individuals [62, 63].

To facilitate reaching out to community groups and networks, cultural community ambassadors and/or mental health champions could be recruited more [64, 65]. These are individuals from ethnic minority community groups who may or may not have used mental health services. In an advocatory role, they can provide informal mental health support and help community members to access services. These have been shown to improve levels of trust between potential service users and services through their understanding of culture and language [64, 66].

Peer support was a need and want for our service users. However, many discussed the perceived lack of commonalities they shared in existing peer groups due to being the only ethnic minority present. Studies have highlighted the discomfort many may feel in social settings when group members are predominantly (or otherwise solely) of White ethnicity [67]. Drawing on the understanding of the impact of peer support and its potential positive effects on mental health outcomes [61, 68], it is recommended that consideration be given to the set-up of peer groups in ethnic minority communities led by mental health champions or supporting peers from an ethnic minority background.

In terms of language, service users who were Kurdish, Arabic, and Bangladeshi encountered difficulties when interacting with interpreters used by SPFT. This was mainly due to differences in dialects and accents and feeling misunderstood which is consistent with other research findings [69]. Researchers suggest that this could have serious consequences as interpreted information is used to decide treatments [70]. It is therefore recommended that the interpreter’s dialect and specific language should match the service user’s whenever possible [71].

Service users expressed feeling overwhelmed at times by the information provided by health care practitioners when they are unwell. This can pose as a barrier to health improvement as the ability to retain and absorb information can be limited [72]. Therefore, it is recommended that when providing information, health professionals should be guided by the service user in terms of pace, timing and delivery.

Service users described experiencing trauma because of their culture or ethnicity either as an individual or as part of an ethnic minority community group. For instance, childhood bullying and racial discrimination were seen by a few as having long-lasting negative effects into adulthood. This is supported in the literature which discusses socioemotional and wellbeing difficulties in adulthood following childhood bullying [44, 73–75]. It was felt that the impacts of these experiences were not well recognised by health practitioners. In general, service users recommended practitioners receive more training in cultural awareness and culturally adapted therapies. This is consistent with previous studies highlighting the need for culturally sensitive, competent, and compassionate care [20, 64, 75, 76]. Recognising this, one London-based NHS trust is already offering a “Traumas of Racism” course [77] in collaboration with their Recovery College [78]. This may help improve service provider’s understanding of how deeply rooted the impacts can be. Enhanced knowledge and skills around different cultural contexts not only lead to better therapeutic relationships, linking to improved health outcomes [64], but may also help healthcare professionals feel better equipped to deal with emotionally difficult situations [79].

Finally, some felt that there should be more mental health awareness and support offered through schools. This aligns with the literature which indicates that children and young people from ethnic minority communities are at higher risk of experiencing trauma compared to their peers, but are least likely to access mental health care [80]. The school setting, therefore, would be a convenient location to provide much needed support. Encouragingly in spring 2022, under the NHS Long Term Plan (2019) [81], there were 287 Mental Health Support Teams (including SPFT) operating in over 4,700 schools and colleges in England [82]. Future assessments of the programme’s outcomes and impact should take these disparities into account. For instance, a measure of success should be an increase in numbers of children and young people from ethnic minority communities accessing their services. Futher more, patterns of service use from different community groups should be in line with UK population figures. These could be seen as positive steps towards reducing mental health inequalities for these groups with potential impacts into adulthood.

### Limitations

The purpose of this service evaluation was to work together with service users, SPFT staff, and stakeholders to design a project to explore ethnic minority community service users’ experiences. As this was a qualitative project without random selection or stratification, we cannot claim to have included a random or representative sample of participants. We therefore cannot generalise our findings beyond SPFT services. Despite this, the service user narratives may be helpful for gaining an understanding of some experiences and planning other services.

### Recommendations for the future

Overall, the findings from this service evaluation led to seven key recommendations for SPFT which are summarised in Table 3. Due to the commonalities among specialist mental health services, these recommendations are likely to be applicable to other similarly organised community health services.

**Table 3:** Service users’ recommendations for SPFT

1. Increase awareness of SPFT mental health services, particularly in those from lower socio-economic groups (education, social class and occupation of the individual).
2. Increase the number of cultural community ambassadors/mental health champions and supporting peers from different ethnic minority groups within the Trust.
3. Increase availability of online mental health support as an alternative method to face-to-face sessions.
4. Cultural training for mental health staff especially to improve understanding of cultural traumas faced by some ethnic minority communities across the life course.
5. Work with interpreter providers to match more closely the language and dialect of interpreters with the service user they are supporting.
6. Training staff around information delivery and sensitivity preventing emotional overload and distress.
7. SPFT to have more presence in schools to help children with their mental health.

## Conclusion

This service evaluation is important for highlighting how COVID-19 has impacted provision and access to SPFT specifically for ethnic minority service users. Lockdown impacted face-to-face appointments and many therapy services were moved online. Some service users benefited from having online sessions, emphasising the potential inclusiveness of this method. However, for some, multiple occupancy and multigenerational households made privacy difficult. Mental health advocates from ethnic minority backgrounds were recommended to help encourage more people from their communities to come forward to seek support for their mental health difficulties. This was seen to be beneficial in communities where mental health is not often talked about. Some recalled traumatic events from their childhood, which included discrimination and bullying, and described its long-lasting negative impacts. Due to the social context of COVID-19, the feelings of trauma resurfaced for a few. However, it was then difficult to access the timely mental health support they needed. Some described the need for more cultural training for health practitioners. Service users provided many recommendations relevant for reducing inequalities for ethnic minorities. Some focused on improving services for ethnic minority communities, and some which could consequently impact all.

### Electronic supplementary material

Below is the link to the electronic supplementary material.

Supplementary Material 1

## Data Availability

A de-identified version of the dataset used and/or analysed during the current study is available from the corresponding author on reasonable request. The interview schedule is available in the supplementary materials.
