# Supplementary material for: A co-produced service evaluation of ethnic minority community service user experiences of a specialist mental health service during the COVID-19 pandemic
Source: BMC Health Serv Res. 2023 Oct 17;23:1107. doi: 10.1186/s12913-023-10115-4 (PMC10583414; doi:10.1186/s12913-023-10115-4)
Supplement: Supplementary file 1 — Supplementary Material 1 [file 12913_2023_10115_MOESM1_ESM.docx]

# Supplementary Materials

## Supplementary Material 1: Recruitment Poster


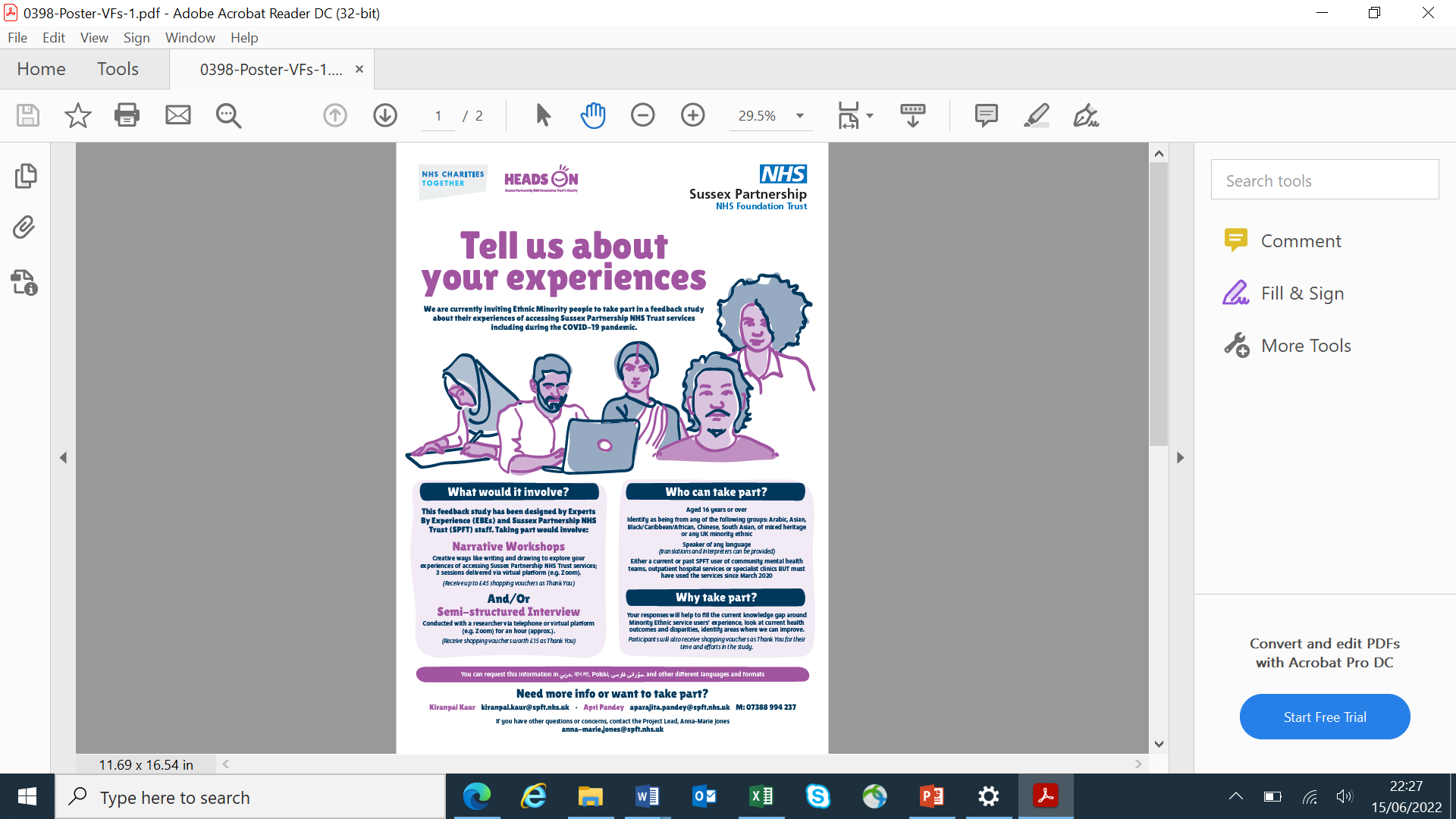


## Supplementary Material 2: Interview schedule

***The mental health journey***
1. I thought we could talk a little bit about your mental health journey and how it led you to SPFT.
How did your mental health journey begin? [timeline]
*[your journey could mean from the point where you sought help,* *interacting with clinical service, to seeing your doctor & any referrals // how did it make you feel // did you access other SPFT services for mental health support]*

2. Do you think that people from ethnic groups are aware of the range of mental health services that are offered at SPFT?

3. Do you think your mental health journey may have been different had you been white or from a different ethnic background?

***COVID-19***
In this part of the interview, we will be talking about COVID-19 - This includes restrictions, SPFT access and increase in racism during the pandemic.
1. Research has shown that lockdown guidelines such as social distancing, shielding, limited person contact has contributed to anxiety and fear in people. What are your thoughts on this and how did these restrictions impact you and your mental health? *[ How did the pandemic affect your access to your doctor? CPN, or any SPFT services?
what happened? // how did it make you feel? // is COVID still affecting your access?]*

2. There have been studies which have shown that there has been an increase in racism and discrimination during COVID, did you ever experience this?
*[did you experience it at SPFT?]
[positive BLM movement; Chinese hate crimes; East Asian descent; South Asian descent]*

***Fairness, Equality & Discrimination***
In these sets of questions, we will be exploring your take on racism, fairness, equality and discrimination.

1. What does being treated with fairness and equality mean to you?
[what does discrimination mean to you]

2. Has there been a situation where you were treated differently to a friend/family member who is from a different ethnic/racial background to yours?
*[interracial // colour difference // inter ethnicity] [has this happened at SPFT]*

3. Based on your experiences, what would you recommend for SPFT to do in order to make sure everybody is being treated with fairness and equality?

***Communication***
In this part of the interview, we will be discussing your experiences of communication at SPFT and any recommendation you would like to make to improve this.
1. Communication could mean talking to a practitioner about your diagnosis over the phone, in person or video call. It could also mean receiving appointment letters and leaflets to help guide you through a diagnosis. Do you have any positive or negative experiences with regard to SPFT communication that you'd like to share?

MATERIALS
[In your own experience, are these materials [leaflets, information sheets] easy to understand in terms of language and culture? [are they readily available]

2. Has there been a time at SPFT where you have not been listened to or heard by us?
*[this could mean at reception, in consultations or at follow-ups]*

3. Would you like to make any recommendations to help mental health services and the trust to communicate better with you and the general minority ethnic community? [This can include appointment reminders, your consultations and the trust's materials.]

***Culture***
We will be talking about culture and its importance to you.
-For me, culture is an integral part of who I am. It's part of my identity. I am British and Indian, bilingual in Punjabi. I feel that I have a 50/50 split with how I identify as myself, I like listening to English music and Bollywood music eating English foods in the same way that I like eating Indian foods. For me, they complement one another. For others it may be less balanced in that they might connect more or less strongly with their heritage than ‘being English’

1.What does culture mean to you?
*[values // principles // upbringing // background // faith // food // religion // customs // beliefs and attitudes // immigration // arts // language // dress // greetings // sex differences]*

2. How important is it to you for health professionals to understand your culture and background?

3. Do you or somebody that you know have any experience of using the language interpretation services during consultations?
*[what was the experience like]
[do you remember how the translator was matched to your needs? Religion, ethnicity, language dialects, sex, and maybe caste/class]*

4. Did the services meet your hopes and expectations?

5. If you could improve anything about the interpretation service, what would it be?

***Stigma***
1. There is research which shows that stigma about mental health remains a problem within all communities and also ethnic groups. It may even cause for people not to reach out for help when they are in need of it the most. How do you think SPFT can help an individual access our mental health services when it might be a problematic area within their community?

2. Have you ever felt that you were stigmatised, frowned upon or excluded, because of your mental health condition and/or ethnicity? Have you ever not spoken to your family or friends about your mental health condition –if so why?

***Final SPFT feedback***

Finally, do you have any more feedback for SPFT mental health services? Things we could improve or make more accessible to everybody?

Thank you for your time, your response has been valuable to us all.
